# Pathway‐based protein–protein association network to explore mechanism of *α*‐glucosidase inhibitors from *Scutellaria baicalensis* Georgi against type 2 diabetes

**DOI:** 10.1049/syb2.12019

**Published:** 2021-04-26

**Authors:** Le Wang, Wenbo Diwu, Nana Tan, Huan Wang, Jingbo Hu, Bailu Xu, Xiaoling Wang

**Affiliations:** ^1^ Key Laboratory of Phytochemistry College of Chemistry and Chemical Engineering Baoji University of Arts and Sciences Baoji China; ^2^ College of Computer Science and Technology Baoji University of Arts and Sciences Baoji China; ^3^ College of Electronic and Electrical Engineering, College of Chemistry and Chemical Engineering Baoji University of Arts and Sciences Baoji China

## Abstract

Natural products have been widely used in the treatment of type 2 diabetes (T2D). However, their mechanisms are often obscured due to multi‐components and multi‐targets. The authors constructed a pathway‐based protein–protein association (PPA) network for target proteins of 13 α‐glucosidase inhibitors (AGIs) identified from *Scutellaria baicalensis* Georgi (*SBG*), designed to explore the underlying mechanisms. This network contained 118 nodes and 1167 connections. An uneven degree distribution and small‐world property were observed, characterised by high clustering coefficient and short average path length. The PPA network had an inherent hierarchy as *C(k)∼k* ^−0.71^. It also exhibited potential weak disassortative mixing pattern, coupled with a decreased function *Knn* (*k*) and negative value of assortativity coefficient. These properties indicated that a few nodes were crucial to the network. PGH2, GNAS, MAPK1, MAPK3, PRKCA, and MAOA were then identified as key targets with the highest degree values and centrality indices. Additionally, a core subnetwork showed that chrysin, 5,8,2′‐trihydroxy‐7‐methoxyflavone, and wogonin were the main active constituents of these AGIs, and that the serotonergic synapse pathway was the critical pathway for *SBG* against T2D. The application of a pathway‐based protein–protein association network provides a novel strategy to explore the mechanisms of natural products on complex diseases.

## 1 INTRODUCTION

The global incidence of type 2 diabetes (T2D) in adults is increasing worldwide [1]. It is a complex disease affected by many factors and complications. In 2020, a detrimental effect of T2D on COVID‐19 was also found [2]. The treatment and management of T2D is becoming increasingly complex [3], and natural products have been considered as the main sources of new drugs [4]. Recently, increasing numbers of natural products have been found to have anti‐T2D properties [5]. Some have been widely used to control diabetes, such as curcumin, flavanone, resveratrol, carotenoid, and polyphenols [6]. The α‐glucosidase inhibitors (AGIs) are described as the most effective anti‐diabetic drugs in the management of T2D [7]. They can suppress the postprandial blood glucose and insulin levels. Many α‐glucosidase inhibitors originate from natural products, especially phytoconstituents [8].

Flavonoids are a group of polyphenols, and are widely distributed in plants [9]. Flavonoids could modulate the activity of enzymes and affect the behaviour of cell systems [10]. A series of flavonoids show antidiabetic properties and activities in the treatment of diabetic complications, including apigenin, hesperidin, catechin, etc. [11]. Some are considered as promising AGIs, such as luteolin, isovitexin, and quercetin [7]. *Scutellaria baicalensis* Georgi (*SBG*) is a widely used medical plant in Asia [12]. Flavonoids and their glycosides are considered to be characteristic components of *SBG* [13]. Extract of this plant has been reported to show an α‐glucosidase inhibition activity [5]. In the authors’ previous works [14], a total of 32 flavonoids from the root of *SBG* were extracted and identified. Moreover, 13 of them were exhibited as α‐glucosidase inhibitors, including wogonin, chrysin, and oroxylin A, etc. Their contributions to the bioactivities of *SBG* were also investigated. However, more works should be done to elucidate the molecular mechanisms of these flavonoids against T2D.

Interactions between individual agents determine the structures and functions of many biological systems [15]. It is noteworthy that the associations between target proteins contribute much to modulate cellular physiology and expand the opportunity for drug discovery [16]. The protein–protein interaction profiles are extremely important to the pharmacological effects of natural products [17]. Nevertheless, systematic analyses of these interrelationships are still challenging tasks. One of the primary reasons for this is that natural products have chemical diversity and the ability to interact with multi‐targets [18]. To fully understand their pharmacological effects, it is vital to explore the associations between all the target proteins for bioactive constituents in natural products [19]. Additionally, a global methodology is needed to extract the related active constituents and biological pathways.

In recent years, complex network theory has been applied to the drug development strategies [20]. It is also known as ‘network pharmacology’ or ‘system pharmacology’ in the research into natural products [21]. This approach aims to pick up information from big data, and summarise rules of individual parts. It is suitable for extracting biological information from large amounts of chemical or biological data [22]. Many studies have applied complex network methodology to investigate the therapeutic potential of natural products [23]. For instance, a network pharmacology‐based analysis found that *Rhizoma coptidis* played an anti‐diabetic role mainly via hormone receptor activity, glutathione binding, steroid binding, etc. [24]. Another study constructed a component/target/pathway network for *Rhizoma coptidis* against T2D, and 12 active components, 57 targets, as well as 38 signalling pathways were screened [25]. It suggested significant potential of this tool in predicting pharmacological actions of active ingredients from medicinal plants against T2D. Guo et al. applied the network methodology to explore the effects of *Gynura procumbens* (*Lour*.) on T2D, and revealed that the PI3K/Akt signalling pathway played a momentous role [26]. Patil et al. investigated the molecular mechanisms of action of 11 common herbs used for the management of T2D, using molecular docking, gene set enrichment analysis, and network pharmacology [27]. The network analysis results showed that the PI3K‐AKT signalling pathway was a key pathway of T2D and its complications that was modulated by the phytoconstituents. In summary, the emerging tool of a complex network would greatly contribute to drug discovery.

Herein, the authors used a complex network model to investigate the underlying mechanisms of AGIs from *SBG* against T2D. Target proteins of these AGIs were organised into a pathway‐based protein–protein association (PPA) network. The network architectures were studied in a systematic manner. Statistical and topological analyses were performed to investigate the interrelationships between nodes. Furthermore, a series of parameters were calculated to identify key nodes in the network, which indicated the main active constituents, key targets and critical biological pathways for the AGIs from *SBG* against T2D.

## 2 MATERIALS AND METHODS

In the authors’ previous studies [14], 13 α‐glucosidase inhibitors (AGIs) were identified from *SBG* by ultrafiltration LC‐MS method, including tenaxin I, skullcapflavone II, viscidulin III, etc. Detailed information about these compounds is listed in Table S1. They were organised as a chemical ingredients database for the next network analysis.

Target proteins of the AGIs were collected from SuperPred (http://prediction.charite.de/) and DrugBank (https://www.drugbank.ca/). Target prediction for the input compounds was also performed by SuperPred, based on the similarity distribution among ligands [28]. Information of these proteins was made uniform by the universal protein resource (Uniprot, http://www.uniprot.org/). Pathway analysis was applied to these target proteins using the Database for Annotation and Integrated Discovery (DAVID 6.8, https://david.ncifcrf.gov/). Raw *p*‐values were adjusted with the Benjamini & Hochberg procedure (*p* < 0.05) [29]. Moreover, the pathways containing only one or two proteins were excluded from the results. An association was established between two proteins if they were both involved in one or more pathways.

Complex network methodology was employed to study the interrelationships between the target proteins of AGIs from *SBG*. A pathway‐based protein–protein association network was then constructed. The PPA network contained many nodes and edges, in which nodes referred to the target proteins, and edges represented associations between nodes. This network was visualised by Pajek (Version 5.1, Batagelj and Mrvar). A set of parameters were investigated for further interpretation of the PPA network, using MATLAB 2016a (The MathWorks Inc.).

## 3 RESULTS

### 3.1 Construction of the pathway‐based protein–protein association (PPA) network

A total of 13 potential AGIs were identified from *SBG* by ultrafiltration UPLC‐Q‐TOF in the authors’ previous experiments. These compounds were organised as a chemical ingredients database (Table S1), containing viscidulin III, chrysin‐7‐O‐*β*‐D‐glucopyranoside, skullcapflavone II, 2′,6′,7‐trihydroxy‐5‐methoxyflavanone, etc. Herein, a series of 118 targets were collected using web tools (Table S2). Parts of these proteins were therapeutic targets of T2D [30], such as glucocorticoid receptor, peroxisome proliferator‐activated receptor delta, poly(ADP‐ribose) polymerase 1, etc. Numerous targets suggested that AGIs from *SBG* were involved in various signal pathways.

A pathway contains a set of cascade reactions among numerous biomolecules. It regulates various biological functions in the organism. The target proteins of AGIs from *SBG* were involved in 86 pathways (Table S2), including the oestrogen signalling pathway, ascorbate and aldarate metabolism, thyroid hormone signalling pathway, pentose and glucuronate interconversions, etc. Abnormal pathways mean perturbations in the intracellular or intercellular network between tissues and organs.

**FIGURE 1:**
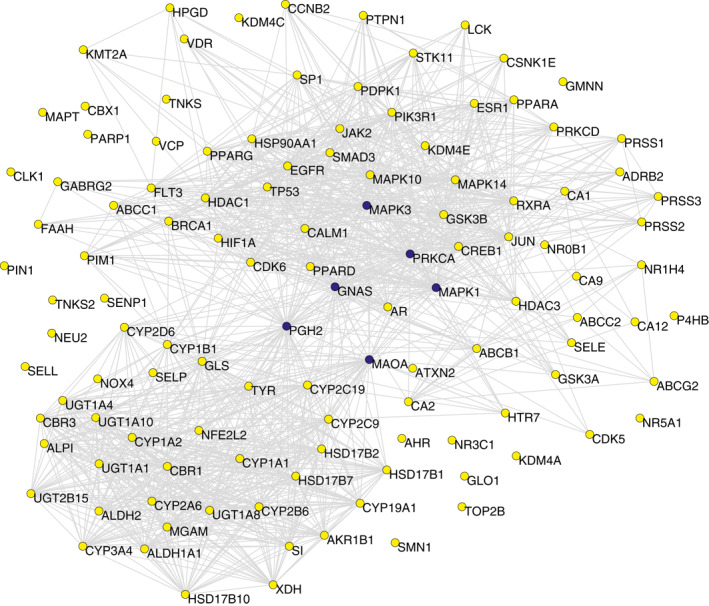
Pathway‐based protein–protein association (PPA) network of α*‐*glucosidase inhibitors (AGIs) from *Scutellaria baicalensis* Georgi (*SBG*), containing 118 nodes and 1167 edges. Nodes indicate the target proteins of AGIs from *SBG*. Edges represent associations between a pair of proteins. The blue nodes represent key targets exhibiting the highest values of degree and centrality indices in the PPA network

Interactions between target proteins are one of the core processes for the effects of natural products on cells. A pathway‐based protein–protein association network was then constructed to investigate interrelationships for targets of AGIs. This network has 118 nodes (*N* = 118) and 1167 connections (*E* = 1167). As shown in Figure 1, a few nodes were highly connected with others, whereas many others were less connected or even isolated. This indicated that the nodes had different significances in PPA network. The authors made further investigation of the topological parameters of the PPA network to explore the behaviours of these target proteins.

### 3.2 Properties of the PPA network for α‐glucosidase inhibitors from *Scutellaria baicalensis* Georgi

The global properties of systems are always determined by the overall framework rather than individual parts. A complex network provides an approach to get information by calculating the network parameters, which contain lots of biological information, and could help to interpret the network locally [31].

#### 3.2.1 Diameter and average path length

Diameter (*D*) indicates the maximum distance between each group of nodes. Average path length (*L*) represents the mean distance over all pairs of nodes.

(1) $$D=\text{max}\left\{d_{\mathrm{ij}}\right\}$$

(2) $$L=\frac{1}{N\left(N-1\right)}\sum_{i\neq j}d_{\mathrm{ij}}$$

*N* is the total number of nodes in the PPA network, and *d*_*ij*_ is the shortest path length from a node *i* to *j*. The connected components of the PPA network showed a very short average path length (*L* = 1.92), smaller than a random network with the same amount of nodes. Additionally, the diameter of this network was 4. This meant that there were at most four links between any pair of nodes. Therefore, target proteins of AGIs from *SBG* appeared to be tightly linked with each other. This further confirmed that the α‐glucosidase inhibitors from *SBG* worked through multi‐targets.

#### 3.2.2 Clustering coefficient

The clustering coefficient reflects the cohesiveness of neighbours for a node, which measures the trend of nodes to form connected triangles. *C* of a node *i* (*C*_*i*_) is displayed below:

(3) $$C_{i}=\frac{2e_{i}}{k_{i}\left(k_{i}-1\right)}\left(k_{i}\geq 2\right)$$

This parameter goes from zero to one. When *C*_*i*_ inclines to zero, the node is among the unintegrated clusters or part of a loosely connected group. Conversely, the node is centred in a highly interconnected cluster. The clustering coefficient of the whole network (*C*) is the average *C*_*i*_ of all nodes. It shows the trend of nodes to be involved in clusters. *C*(*k*) reflects the distribution of clustering coefficient for all the nodes.

**FIGURE 2:**
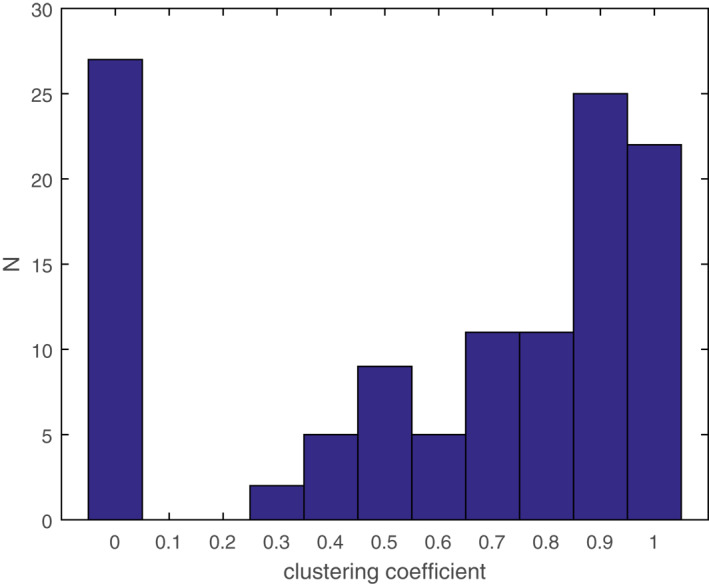
Distribution of clustering coefficient to show the proportion of nodes with a specific *C*_*i*_ value

The clustering coefficient of the PPA network was 0.83. It showed a strong tendency of these proteins to form clusters. The distribution of *C*_*i*_ was analysed in Figure 2. Apparently, most of the non‐isolated nodes had a high *C*_*i*_ value that was larger than 0.5. It was probable for these nodes to be involved in a more connected cluster, indicating that a critical pathway existed for the bioactivities of α‐glucosidase inhibitors from *SBG*.

Many networks in the real world are either completely regular or completely random. However, the PPA network demonstrated a small‐world property, characterised by a high clustering coefficient and short average path length [32]. This suggested that the network was composed of many small, closely linked, hierarchical clusters, and presented as large, less cohesive cells. Disturbances on a few key nodes would diffuse rapidly into the whole network. In other words, a small number of key targets were crucial to the bioactivities of α‐glucosidase inhibitors from *SBG*. This feature also existed in many other biological networks [33].

**FIGURE 3:**
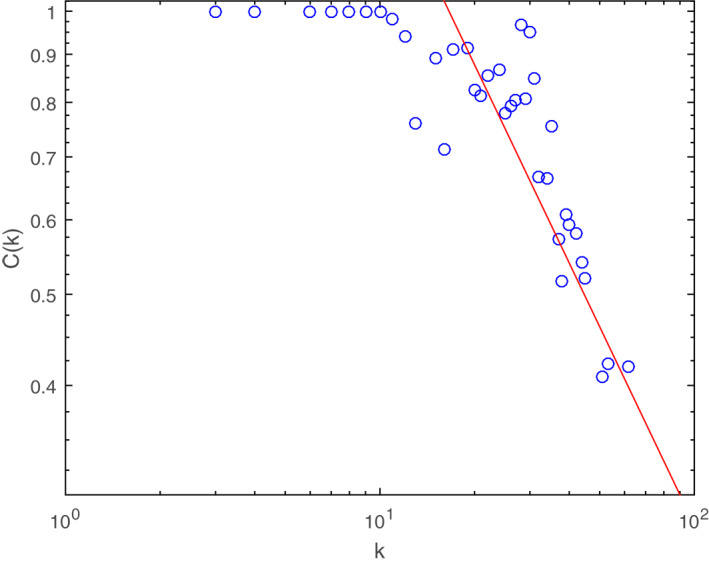
The average clustering coefficient for the nodes in PPA network

*C(k)* was evaluated and fitted in Figure 3. It showed a power‐law decay with an exponent of 0.71*, C(k)∼k* ^−0.71^. The PPA network appeared as a hierarchical system for the non‐uniform, power‐law of *C(k)*. It indicated that many nodes tended to be involved in heavily connected regions, which produced a higher clustering coefficient of the network. In other words, many targets of AGIs were strongly connected, and simultaneously belonged to a few pathways. These interconnections might play an important part in the pharmacological effects of AGIs from *SBG*.

#### 3.2.3 Degree correlation and assortativity

Degree (*k*) is one of the most important characteristics for a node. The number of direct links for node *i* is defined as *k*_*i*_. The mean value of all *k*_*i*_ is the average degree 〈*k*〉 of the network. The nodes with the most links are defined as hubs of the network. Degree distribution, represented as *P(k)*, describes the proportion of nodes with a particular number of links.

(4) $$k_{i}=\sum_{j=1}^{N}e_{\mathrm{ij}}$$

(5) $$<k>=\frac{1}{N}\sum_{i=1}^{N}k_{i}$$

where *e*_*ij*_ is the number of links from node *i* to *j*.

Degree correlation measures the influence of connectivity for a node on its neighbours [34]. Degree correlations of the network are represented as the average nearest neighbours degree *K*_*nn,i*_ for node *i*. *K*_*nn*_(*k*) indicates *K*_*nn*_ of nodes with a degree *k*.

(6) $$K_{nn,i}=\frac{1}{k_{i}}\sum_{j\in N_{i}}a_{ij}k_{ij}$$

The network is either assortative or disassortative, determined by *K*_*nn*_(*k*) is increasing or decreasing as a function of *k*. If no correlation exists among all nodes, *K*_*nn*_(*k*) is 0.

The assortativity coefficient (*r*) measures degree correlations between neighbours in a network [35]. If *r* is a positive value, the network is assortative, otherwise it is disassortative with a negative value of *r*.

(7) $$r=\frac{E^{-1}\sum_{i}x_{i}y_{i}-{\left[E^{-1}\sum_{i}\frac{1}{2}\left(x_{i}+y_{i}\right)\right]}^{2}}{E^{-1}\sum_{i}\frac{1}{2}\left(x_{i}^{2}+y_{i}^{2}\right)-{\left[E^{-1}\sum_{i}\frac{1}{2}\left(x_{i}+y_{i}\right)\right]}^{2}}$$

**FIGURE 4:**
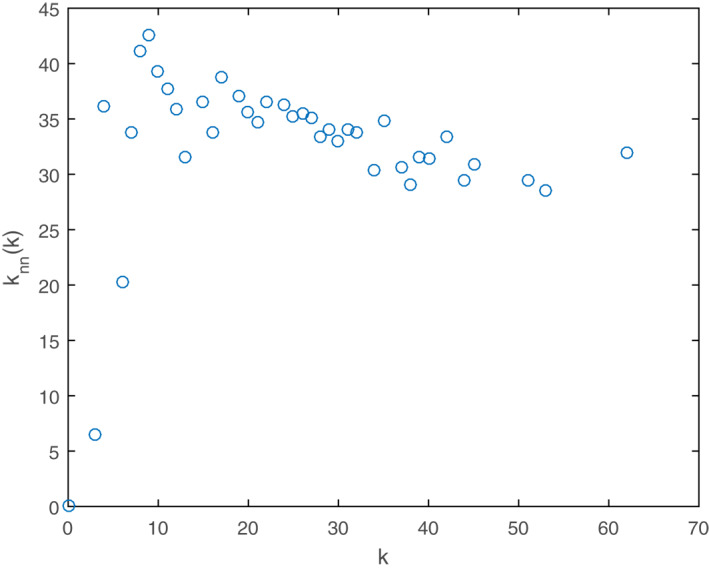
The average nearest neighbours degree for the nodes in the PPA network

The average nearest neighbours degree, *K*_*nn*_ (*k*) of the PPA network is shown in Figure 4. It is exhibited as a decreasing function of *k*, as well as a potential weak disassortative mixing (*k* > 10). The assortativity coefficient (*r*) of the PPA network was −0.1, showing the same trend as *K*_*nn*_. This illustrated that interactions might exist between nodes with large degrees and small degrees. Many biological networks are also inclined to be disassortative [36]. This might be the result for the highly complex constitution of organisms.

### 3.3 Hubs, central nodes of the PPA network, and key targets for AGIs from SBG

#### 3.3.1 Hubs of the PPA network

Numerous nodes in the network have different responsibilities. Degree distribution *P(k)* reflects the diversity of a network. The numbers and frequencies of different degree values are listed in Table 1.

The PPA network showed an uneven degree distribution. A part of nodes was isolated or had small degrees less than 10, whereas that of a few nodes were larger than 50. Average degree 〈*k*〉 of the PPA network was 19.76, implying that an average of nearly 20 targets appeared in common pathways.

**FIGURE 5:**
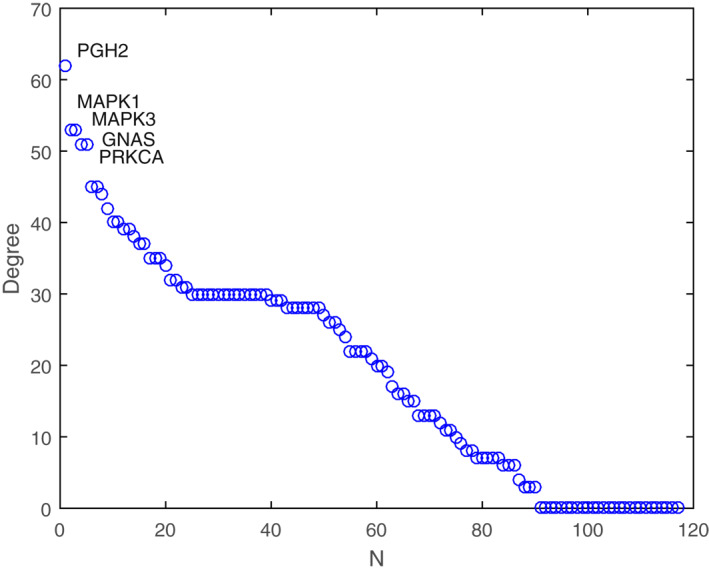
Degree of all nodes shown in descending order

The most highly connected nodes are defined as hubs. All nodes were sorted according to degree values (Figure 5). Among the 118 targets of AGIs from *SBG*, prostaglandin G/H synthase 2 precursor (PGH2) had the highest degree of 63, then mitogen‐activated protein kinase 1 (MAPK1, *k* = 53), mitogen‐activated protein kinase 3 (MAPK3, *k* = 53), guanine nucleotide‐binding protein G(s) subunit alpha (GNAS, *k* = 51) and protein kinase C alpha type (PRKCA, *k* = 51). The five proteins showed much higher degree values than the average (〈*k*〉 = 19.76), and were considered as hub nodes of the PPA network. Hubs are always located to determine the network function [32]. Larger degrees demonstrated that these targets had larger impacts on the network. Although natural products had many target proteins, the highly connected ones involved in various pathways might contribute most to the pharmacological effects.

**TABLE 1:** Degree distribution of the pathway‐based protein association network

*k*	Count^1^	*P(k)* ^2^	*k*	Count	*P(k)*	*k*	Count	*P(k)*
63	1	0.85	31	15	12.71	15	2	1.69
53	2	1.69	30	3	2.54	13	4	3.39
51	2	1.69	29	8	6.78	12	1	0.85
46	1	0.85	28	1	0.85	11	2	1.69
45	1	0.85	27	1	0.85	10	1	0.85
44	1	0.85	26	2	1.69	9	1	0.85
43	1	0.85	25	1	0.85	8	2	1.69
40	2	1.69	24	1	0.85	7	5	4.24
39	2	1.69	22	4	3.39	6	3	2.54
38	1	0.85	21	1	0.85	4	1	0.85
37	2	1.69	20	2	1.69	3	3	2.54
36	3	2.54	19	1	0.85	0	27	22.88
34	1	0.85	17	1	0.85			
32	3	2.54	16	2	1.69			

^1^ Number of the occurrence of a degree value.

^2^ Percentage of nodes with a certain degree value in all nodes (%).

#### 3.3.2 Central nodes of the PPA network

Centrality demonstrates the relative influence of a node on the network structure. Three centrality indices (*CI*), degree centrality (*C*_*d*_), betweenness centrality (*C*_*b*_), and closeness centrality (*C*_*c*_) are evaluated to search central nodes of the PPA network. *C*_*d*_ indicates the proportion of other nodes adjacent to a node. *C*_*b*_ is the total quantity of the shortest paths through a node. *C*_*c*_ is the quantity of other nodes divided by the sum of distances between one node and all the others. The equations are as follows:

(8) $$C_{d}=\frac{k_{i}}{N-1}$$

(9) $$C_{b}=\sum_{j\left(<k\right)}^{N}\sum_{k}^{N}\frac{g_{\text{jk}}\left(i\right)}{g_{\text{jk}}}$$

(10) $$C_{c}=\frac{N-1}{\sum_{j=1}^{N}d_{\text{ij}}}$$

where *g*_*jk*_ is the number of geodesics connecting nodes *j* and *k*, and *d*_*ij*_ is the shortest path length between nodes *i* and *j*.

**FIGURE 6:**
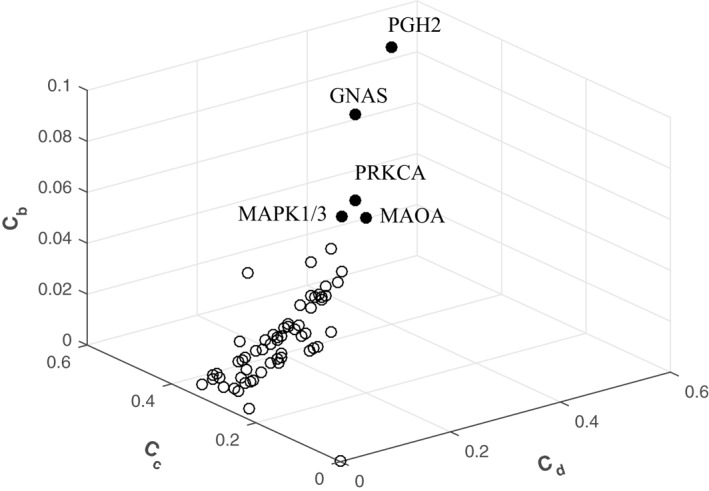
Three‐dimensional diagram to show the centrality indices (*CI*) values of all nodes in the PPA network

The central locations of a network are more important than marginal or isolated positions [37]. The central nodes of the PPA network were investigated by three indexes. Figure 6 is a 3D graph illustrating the distribution of *CI*. The integration of *CI* seemed approximately uniform. However, some abnormal values were exhibited as outliers, including PGH2 (*C*_*d*_ = 0.538, *C*_*b*_ = 0.090, *C*_*c*_ = 0.578), GNAS (*C*_*d*_ = 0.436, *C*_*b*_ = 0.069, *C*_*c*_ = 0.538), MAPK1 (*C*_*d*_ = 0.453, *C*_*b*_ = 0.028, *C*_*c*_ = 0.534), MAPK3 (*C*_*d*_ = 0.453, *C*_*b*_ = 0.028, *C*_*c*_ = 0.534), PRKCA (*C*_*d*_ = 0.436, *C*_*b*_ = 0.036, *C*_*c*_ = 0.538), and monoamine oxidase type A (MAOA, *C*_*d*_ = 0.393, *C*_*b*_ = 0.036, *C*_*c*_ = 0.507). MAPK1 and MAPK3 had the same *CI* values, which are overlapped in Figure 6. Detailed data of *CI* are listed in Table S3.

#### 3.3.3 Key targets for AGIs from *SBG*

The six central nodes were located in key positions of the PPA network. A total of 70 neighbours were directly linked to them. These target proteins accounted for 59.3% of all nodes, and were involved in common pathways with the central nodes. Moreover, five of the central nodes were also hubs of the PPA network. High connectivity and centrality suggested that disturbances to the six proteins would spread rapidly throughout the whole network. Therefore, they were considered as key targets for the AGIs from *SBG*.

Recent studies have demonstrated the association between these key targets and T2D. PGH2 generates prostaglandins and causes insulin insensitivity. PGH2 polymorphisms were found to play a role in mediating susceptibility to T2D in Pima Indians [38]. The GNAS gene encodes the heterotrimeric Gs protein α‐subunit. It is an important regulator of insulin secretory capacity in pancreatic β‐cells [39]. MAPK1 and MAPK3 belong to the MAPK/ERK cascade, which could affect insulin signalling [40]. They are increased in human and rodent adipose tissue in diabetic states [41]. PRKCA encodes a cytoplasmic serine/threonine kinase. Variants in PRKCA are significantly associated with diabetes [42]. MAOA is a critical regulator of neurotransmitter signalling at monoaminergic synapses. Polymorphisms in MAOA were found to be associated with obesity, a key factor contributing to the incidence of T2D [43]. These reports further confirmed the importance of the key targets in the treatment of T2D.

### 3.4 Core subnetwork, main active constituents, and critical pathways for type 2 diabetes

**FIGURE 7:**
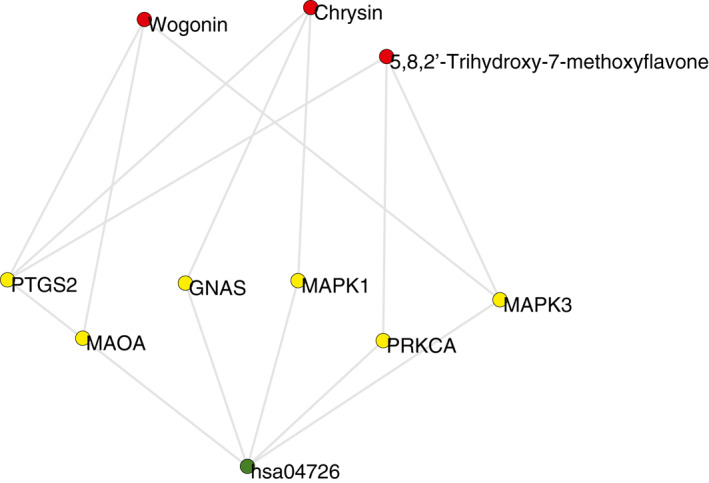
Core subnetwork of α*‐*glucosidase inhibitors (AGIs) from *Scutellaria baicalensis* Georgi (*SBG*). Red nodes indicate the main active constituents, yellow nodes are the key targets, and the green node represents the critical pathway

T2D is a complex disease regulated by a group of pathways [44]. Accordingly, the mechanism of AGIs from *SBG* in T2D is confused. Although the number of key targets was small, these proteins played a significant part in the pharmacological effect of AGIs. To enhance the interpretation, the key targets together with related AGIs, pathways were extracted. These data were then integrated into a core subnetwork, containing 10 nodes and 15 connections (Figure 7).

These nodes had significant impacts on the global function of the PPA network. The node information is listed in Table 2.

**TABLE 2:** Information on the nodes in the core subnetwork

Main active constituents	CAS^1^	Key targets	Recommended name^2^	Critical pathways	Entry^3^
Chrysin	480‐40‐0	PGH2	Prostaglandin G/H synthase 2	Serotonergic synapse pathway	hsa04726
Wogonin	632‐85‐9	GNAS	Guanine nucleotide‐binding protein G(s) subunit alpha isoforms short		
5,8,2′‐Trihydroxy‐ 7‐methoxyflavone	77056‐20‐3	MAPK1	Mitogen‐activated protein kinase 1		
		MAPK3	Mitogen‐activated protein kinase 3		
		PRKCA	Protein kinase C alpha type		
		MAOA	Amine oxidase (flavin‐containing) A		

^1^ Registry number (Chemical Abstracts Service) of the α‐glucosidase inhibitors in the core subnetwork.

^2^ Name of protein targets in the core subnetwork, originated from Uniprot.

^3^ KEGG entry of pathway in the core subnetwork.

Three AGIs showed direct associations with the key targets, including chrysin, 5,8,2′‐trihydroxy‐7‐methoxyflavone, and wogonin. They were considered as the main active constituents. Chrysin is a natural component extracted mainly from plants. It has been demonstrated to have a potent antidiabetogenic effect. Chrysin could improve diabetes in streptozotocin‐induced diabetic rats [45]. This compound was also found to ameliorate diabetes‐associated cognitive deficits in Wistar rats [46]. Wogonin has been shown to be effective in controlling diabetes and its complications. It could increase GLUT4 (Glucose transporter 4) trafficking to plasma membrane, which allows increased entry of glucose and thus alleviates hyperglycaemia [47]. The three AGIs from *SBG* are all flavonoids. Bioactivities of flavonoids are dependent on the hydroxylated phenolic structure [48]. Although the reports about pharmacological action of 5,8,2′‐trihydroxy‐7‐methoxyflavone are few, it has a potential antidiabetogenic effect, which should be tested in the future. These main active constituents might contribute most to the pharmacological effects of AGIs from *SBG*.

**FIGURE 8:**
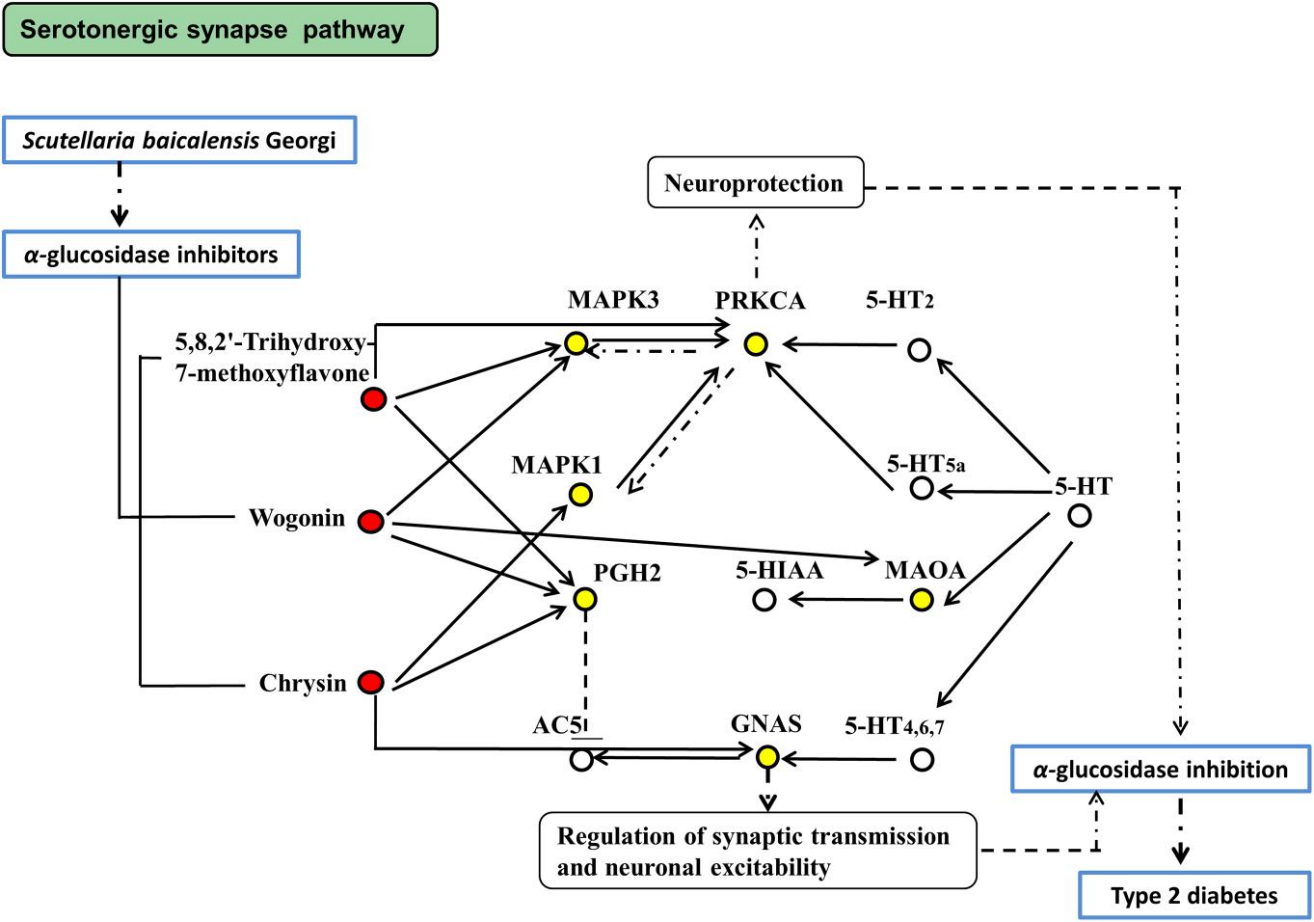
Critical pathway (serotonergic synapse, hsa04726) for α*‐*glucosidase inhibition of *Scutellaria baicalensis* Georgi. Yellow nodes indicate six key targets of the PPA network. Red nodes represent the main active constituents from *SBG* targeted to the six key proteins

All six key targets were involved in the serotonergic synapse pathway (hsa04726). It was exhibited as a critical pathway for *SBG* and T2D (Figure 8). The regulating effects of natural products against T2D are based on various targets and signal pathways [5]. The serotonergic synapse pathway is mainly related to the nervous system, and T2D is a systemic disorder affected by both the central and peripheral nervous systems [49, 50]. Serotonin, also known as 5‐hydroxytryptamine (5‐HT), is a monoamine neurotransmitter. It plays a significant role in many major risk factors for T2D, such as obesity, glucose control, and insulin resistance [51]. Serotonin was reported to control the glucose homeostasis of the nervous system [52]. The serotonin transporter was found to play a potential role in antidepressant‐induced type 2 diabetes [53]. Moreover, serotonin 2C receptor agonists could increase glucose tolerance and improve T2D [54]. Recent research has also identified genetic markers involved in the serotonergic synapse pathway and T2D using a systems biology approach [55]. These reports supported the hypothesis that the α‐glucosidase inhibitors from *SBG* contributed to control T2D through the serotonergic synapse pathway, which needs to be confirmed in future studies.

## 4 DISCUSSION

Natural products are characterised by multi‐components and multi‐targets, which cause difficulties in the mechanism research [56]. The complex network method enables the extraction of information from protein–protein interactions data, and is suitable for exploring the underlying mechanism from a system point of view. Most studies into protein–protein interactions of natural products used major public databases as data sources. For instance, Hu et al. built a human protein–protein interaction network and the T2D disease protein interaction network [57], designed to provide new effective combinations of herbal medicines for T2D. The data were collected from seven databases, including BioGRID, BIND, DIP, HPRD, iRefWeb, IntAct, and MINT. Ren et al. constructed a protein–protein interaction network for Anshen essential oil based on the STRING database, and found that SLC4A4 was in the centre of the targets, followed by HTR3A, HTR2A, DRD2, etc. [58]. In the authors’ previous works [14], they conducted a network analysis of the targets of AGIs from *SBG* and that of commercial drugs for T2D. The interaction data were also calculated by the STRING database. These data mainly originated from experimental data and literature, which focussed on the interrelationships between each pair of proteins. On the other hand, large amounts of targets of natural products are involved in a series of pathways in vivo, and therapeutic effects of natural products are achieved through these signalling pathways [59, 60]. Thus, more attention should be paid to protein interactions based on common pathways. This study aimed to explore mechanisms of AGIs from *SBG* against T2D using a pathway‐based protein–protein association network, which would provide more information from a system point of view. Hub nodes of this network were analysed and extracted, and were considered as key targets, main active constituents, as well as critical pathways for AGIs from *SBG*. These results were also supported by previous reports. However, some important issues to be addressed include that more computational models and experiments are needed to prove these results.

## 5 CONCLUSIONS

In the study discussed herein, a pathway‐based protein–protein association network was built for target proteins of α‐glucosidase inhibitors from *Scutellaria baicalensis* Georgi. This network showed a series of distinct features, such as uneven degree distribution and small‐world property, an inherent hierarchy as *C(k)∼k* ^−0.71^, as well as potential weak disassortative mixing pattern, coupled with decreased function *K*_*nn*_ *(k)* and negative value of assortativity coefficient. These data indicated that the network was greatly affected by a small group of components. PGH2, GNAS, MAPK1, MAPK3, PRKCA, and MAOA were then selected as key targets of these AGIs. The serotonergic synapse was found to be a critical pathway for the AGIs from *SBG* against T2D. These conclusions are also strongly supported by previous reports. Generally, the application of a complex network would expand the authors’ views on natural products in the treatment of T2D.

## CONFLICTS OF INTEREST

The authors declare no conflicts of interest.

## Supporting information

Table S1

Table S2

Table S3
