# Supplementary material for: Pathway‐based protein–protein association network to explore mechanism of α‐glucosidase inhibitors from Scutellaria baicalensis Georgi against type 2 diabetes
Source: IET Syst Biol. 2021 Apr 26;15(4):126–35. doi: 10.1049/syb2.12019 (PMC8675860; doi:10.1049/syb2.12019)
Supplement: Supplementary file 1 — Table S1 [file SYB2-15-126-s001.docx]

**Supplemental Table S1** The *α*-glucosidase inhibitors identified from *Scutellaria baicalensis* Georgi.

| **No.** | **Compound ^a^** | ***t*_R_ (min)** | **Formula** | **Measured m/z ^b^** | **Error ^c^ (ppm)** |
| --- | --- | --- | --- | --- | --- |
| 1 | 2',3,5,6',7-Pentahydroxyflavanone | 5.80 | C_15_H_12_O_7_ | 305.0659 | 1.00 |
| 2 | 2',5,6',7-Tetrahydroxyflavane | 9.33 | C_15_H_12_O_6_ | 289.0709 | 0.80 |
| 3 | Chrysin-7-O-*β*-D-glucopyranoside | 9.55 | C_21_H_20_O_9_ | 417.1179 | -0.30 |
| 4 | Viscidulin III | 9.58 | C_17_H_14_O_8_ | 347.0764 | 0.70 |
| 5 | 2',6',7-Trihydroxy-5-methoxyflavanone | 9.69 | C_16_H_14_O_6_ | 303.0859 | -1.40 |
| 6 | Baicalein-7-O-*β*-D-glucopyranoside | 10.79 | C_21_H_20_O_10_ | 433.1133 | 0.90 |
| 7 | Oroxylin A-7-O-*β*-D-glucuronide methyl ester | 17.42 | C_23_H_22_O_11_ | 475.1231 | -0.80 |
| 8 | 5,8,2'-Trihydroxy-7-methoxyflavone | 17.74 | C_16_H_12_O_6_ | 301.0709 | 0.80 |
| 9 | Skullcapflavone II * | 17.91 | C_19_H_18_O_8_ | 375.1077 | 0.70 |
| 10 | Wogonin * | 18.32 | C_16_H_12_O_5_ | 285.0764 | 2.30 |
| 11 | Chrysin * | 18.62 | C_15_H_10_O_4_ | 255.0654 | 0.80 |
| 12 | Oroxylin A * | 18.71 | C_16_H_12_O_5_ | 285.0760 | 0.90 |
| 13 | Tenaxin I | 19.18 | C_18_H_16_O_7_ | 345.0971 | 0.60 |

a, Compounds were identified by the comparison with exact mass (<5 ppm), reference standards (indicated by an asterisk), as well as the MS fragmentation patterns (Ref. [23]); b, Measured m/z of peak [M+H]^+^; c, Mass accuracy between the calculated m/z and measured m/z of peak [M+H]^+^.
