# Supplementary material for: Pathway‐based protein–protein association network to explore mechanism of α‐glucosidase inhibitors from Scutellaria baicalensis Georgi against type 2 diabetes
Source: IET Syst Biol. 2021 Apr 26;15(4):126–35. doi: 10.1049/syb2.12019 (PMC8675860; doi:10.1049/syb2.12019)
Supplement: Supplementary file 2 — Table S2 [file SYB2-15-126-s003.docx]

**Supplemental Table S2** Target proteins and related pathways of *α*-glucosidase inhibitors from *Scutellaria baicalensis* Georgi

| **No.** | **Target** | **Protein Name** ^a^ | | **No.** | **Pathway** ^b^ |
| --- | --- | --- | --- | --- | --- |
| 1 | P05412 | JUN | | 1 | hsa00980 |
| 2 | P36888 | FLT3 | | 2 | hsa00140 |
| 3 | P16220 | CREB1 | | 3 | hsa05204 |
| 4 | P08047 | SP1 | | 4 | hsa00982 |
| 5 | P51843 | NR0B1 | | 5 | hsa00830 |
| 6 | P15121 | AKR1B1 | | 6 | hsa04915 |
| 7 | P07477 | PRSS1 | | 7 | hsa04919 |
| 8 | P35030 | PRSS3 | | 8 | hsa05200 |
| 9 | P07478 | PRSS2 | | 9 | hsa05223 |
| 10 | P07550 | ADRB2 | | 10 | hsa00983 |
| 11 | P35354 | PGH2 | | 11 | hsa05215 |
| 12 | P03372 | ESR1 | | 12 | hsa04913 |
| 13 | P04637 | TP53 | | 13 | hsa04912 |
| 14 | P38398 | BRCA1 | | 14 | hsa04722 |
| 15 | P83916 | CBX1 | | 15 | hsa00040 |
| 16 | P18507 | GABRG2 | | 16 | hsa05231 |
| 17 | O75496 | GMNN | | 17 | hsa05160 |
| 18 | O94925 | GLS | | 18 | hsa04068 |
| 19 | P84022 | SMAD3 | | 19 | hsa05210 |
| 20 | P34969 | HTR7 | | 20 | hsa04726 |
| 21 | P49759 | CLK1 | | 21 | hsa05230 |
| 22 | Q00535 | CDK5 | | 22 | hsa00053 |
| 23 | P04798 | CYP1A1 | | 23 | hsa05214 |
| 24 | O00519 | FAAH | | 24 | hsa05212 |
| 25 | P10275 | AR | | 25 | hsa05164 |
| 26 | Q96RI1 | NR1H4 | | 26 | hsa04917 |
| 27 | P00918 | CA2 | | 27 | hsa05213 |
| 28 | Q00534 | CDK6 | | 28 | hsa04668 |
| 29 | P11511 | CYP19A1 | | 29 | hsa04012 |
| 30 | P10635 | CYP2D6 | | 30 | hsa04071 |
| 31 | P08684 | CYP3A4 | | 31 | hsa04664 |
| 32 | Q02880 | TOP2B | | 32 | hsa04151 |
| 33 | P04150 | NR3C1 | | 33 | hsa04976 |
| 34 | P49841 | GSK3B | | 34 | hsa04728 |
| 35 | Q16665 | HIF1A | | 35 | hsa04916 |
| 36 | Q16539 | MAPK14 | | 36 | hsa04380 |
| 37 | P21397 | MAOA | | 37 | hsa04723 |
| 38 | P33527 | ABCC1 | | 38 | hsa04660 |
| 39 | P16109 | SELP | | 39 | hsa05142 |
| 40 | Q07869 | PPARA | | 40 | hsa05216 |
| **No.** | **Target** | | **Protein Name** ^a^ | **No.** | **Pathway** ^b^ |
| 41 | Q03181 | | PPARD | 41 | hsa04931 |
| 42 | P37231 | | PPARG | 42 | hsa05161 |
| 43 | P16581 | | SELE | 43 | hsa04261 |
| 44 | P14679 | | TYR | 44 | hsa05221 |
| 45 | P06239 | | LCK | 45 | hsa04150 |
| 46 | P11473 | | VDR | 46 | hsa04914 |
| 47 | P47989 | | XDH | 47 | hsa00910 |
| 48 | P15428 | | HPGD | 48 | hsa04370 |
| 49 | P56937 | | HSD17B7 | 49 | hsa00590 |
| 50 | P05091 | | ALDH2 | 50 | hsa00232 |
| 51 | P00352 | | ALDH1A1 | 51 | hsa04921 |
| 52 | P35869 | | AHR | 52 | hsa04024 |
| 53 | Q99700 | | ATXN2 | 53 | hsa04960 |
| 54 | Q9UNQ0 | | ABCG2 | 54 | hsa05205 |
| 55 | P53779 | | MAPK10 | 55 | hsa00380 |
| 56 | P0DP23 | | CALM1 | 56 | hsa05031 |
| 57 | Q92887 | | ABCC2 | 57 | hsa04750 |
| 58 | P00915 | | CA1 | 58 | hsa00860 |
| 59 | O43570 | | CA12 | 59 | hsa05140 |
| 60 | Q16790 | | CA9 | 60 | hsa05218 |
| 61 | P16152 | | CBR1 | 61 | hsa05220 |
| 62 | O75828 | | CBR3 | 62 | hsa04910 |
| 63 | O95067 | | CCNB2 | 63 | hsa05133 |
| 64 | P05177 | | CYP1A2 | 64 | hsa04930 |
| 65 | Q16678 | | CYP1B1 | 65 | hsa05030 |
| 66 | P11509 | | CYP2A6 | 66 | hsa05145 |
| 67 | P20813 | | CYP2B6 | 67 | hsa04621 |
| 68 | P33261 | | CYP2C19 | 68 | hsa00591 |
| 69 | P11712 | | CYP2C9 | 69 | hsa01100 |
| 70 | Q99714 | | HSD17B10 | 70 | hsa05206 |
| 71 | P14061 | | HSD17B1 | 71 | hsa05203 |
| 72 | P37059 | | HSD17B2 | 72 | hsa04510 |
| 73 | Q04760 | | GLO1 | 73 | hsa05202 |
| 74 | P63092 | | GNAS | 74 | hsa04972 |
| 75 | Q03164 | | KMT2A | 75 | hsa04713 |
| 76 | P14151 | | SELL | 76 | hsa04066 |
| 77 | B2RXH2 | | KDM4E | 77 | hsa05034 |
| 78 | P10636 | | MAPT | 78 | hsa05152 |
| 79 | P28482 | | MAPK1 | 79 | hsa05211 |
| 80 | Q16236 | | NFE2L2 | 80 | hsa04310 |
| 81 | P08183 | | ABCB1 | 81 | hsa03320 |
| 82 | P19793 | | RXRA | 82 | hsa04550 |
| 83 | P11309 | | PIM1 | 83 | hsa04662 |
| **No.** | **Target** | | **Protein Name** ^a^ | **No.** | **Pathway** ^b^ |
| 84 | Q9Y3R4 | | NEU2 | 84 | hsa04920 |
| 85 | Q13285 | | NR5A1 | 85 | hsa04620 |
| 86 | Q16637 | | SMN1 | 86 | hsa04520 |
| 87 | P22309 | | UGT1A1 |  |  |
| 88 | Q9HAW8 | | UGT1A10 |  |  |
| 89 | Q9HAW9 | | UGT1A8 |  |  |
| 90 | P22310 | | UGT1A4 |  |  |
| 91 | P54855 | | UGT2B15 |  |  |
| 92 | O95271 | | TNKS |  |  |
| 93 | P27986 | | PIK3R1 |  |  |
| 94 | Q9NPH5 | | NOX4 |  |  |
| 95 | P27361 | | MAPK3 |  |  |
| 96 | Q9H2K2 | | TNKS2 |  |  |
| 97 | O75164 | | KDM4A |  |  |
| 98 | Q9H3R0 | | KDM4C |  |  |
| 99 | P09923 | | ALPI |  |  |
| 100 | Q9P0U3 | | SENP1 |  |  |
| 101 | P55072 | | VCP |  |  |
| 102 | P49674 | | CSNK1E |  |  |
| 103 | P18031 | | PTPN1 |  |  |
| 104 | P49840 | | GSK3A |  |  |
| 105 | O60674 | | JAK2 |  |  |
| 106 | O15530 | | PDPK1 |  |  |
| 107 | P07900 | | HSP90AA1 |  |  |
| 108 | Q13547 | | HDAC1 |  |  |
| 109 | O15379 | | HDAC3 |  |  |
| 110 | Q13526 | | PIN1 |  |  |
| 111 | Q15831 | | STK11 |  |  |
| 112 | P09874 | | PARP1 |  |  |
| 113 | Q05655 | | PRKCD |  |  |
| 114 | P00533 | | EGFR |  |  |
| 115 | P17252 | | PRKCA |  |  |
| 116 | P07237 | | P4HB |  |  |
| 117 | P14410 | | SI |  |  |
| 118 | O43451 | | MGAM |  |  |

a, Names of the target proteins are uniformed by Uniprot; b, Names of the pathways are uniformed by KEGG.
