# Supplementary material for: Pathway‐based protein–protein association network to explore mechanism of α‐glucosidase inhibitors from Scutellaria baicalensis Georgi against type 2 diabetes
Source: IET Syst Biol. 2021 Apr 26;15(4):126–35. doi: 10.1049/syb2.12019 (PMC8675860; doi:10.1049/syb2.12019)
Supplement: Supplementary file 3 — Table S3 [file SYB2-15-126-s002.docx]

**Supplemental Table S3** Centrality indices (*CI*) values of all nodes in the PPA network

| **No.** | **Protein Name** ^a^ | **Degree centrality (*C_d_*)** | **Betweenness centrality (*C_b_*)** | **Closeness centrality (*C_c_*)** |
| --- | --- | --- | --- | --- |
| 1 | JUN | 0.333 | 0.008 | 0.482 |
| 2 | FLT3 | 0.214 | 0.004 | 0.439 |
| 3 | CREB1 | 0.316 | 0.010 | 0.475 |
| 4 | SP1 | 0.188 | 0.002 | 0.386 |
| 5 | NR0B1 | 0.000 | 0.000 | 0.000 |
| 6 | AKR1B1 | 0.248 | 0.000 | 0.390 |
| 7 | PRSS1 | 0.111 | 0.001 | 0.369 |
| 8 | PRSS3 | 0.111 | 0.001 | 0.369 |
| 9 | PRSS2 | 0.111 | 0.001 | 0.369 |
| 10 | ADRB2 | 0.094 | 0.000 | 0.360 |
| 11 | PGH2 | 0.538 | 0.090 | 0.578 |
| 12 | ESR1 | 0.188 | 0.000 | 0.386 |
| 13 | TP53 | 0.342 | 0.011 | 0.485 |
| 14 | BRCA1 | 0.171 | 0.001 | 0.426 |
| 15 | CBX1 | 0.000 | 0.000 | 0.000 |
| 16 | GABRG2 | 0.060 | 0.000 | 0.381 |
| 17 | GMNN | 0.000 | 0.000 | 0.000 |
| 18 | GLS | 0.368 | 0.026 | 0.496 |
| 19 | SMAD3 | 0.231 | 0.002 | 0.445 |
| 20 | HTR7 | 0.077 | 0.000 | 0.394 |
| 21 | CLK1 | 0.000 | 0.000 | 0.000 |
| 22 | CDK5 | 0.034 | 0.000 | 0.373 |
| 23 | CYP1A1 | 0.274 | 0.002 | 0.434 |
| 24 | FAAH | 0.060 | 0.000 | 0.381 |
| 25 | AR | 0.188 | 0.000 | 0.431 |
| 26 | NR1H4 | 0.051 | 0.000 | 0.345 |
| 27 | CA2 | 0.111 | 0.040 | 0.363 |
| 28 | CDK6 | 0.274 | 0.003 | 0.460 |
| 29 | CYP19A1 | 0.265 | 0.002 | 0.431 |
| 30 | CYP2D6 | 0.188 | 0.004 | 0.426 |
| 31 | CYP3A4 | 0.265 | 0.000 | 0.394 |
| 32 | TOP2B | 0.000 | 0.000 | 0.000 |
| 33 | NR3C1 | 0.000 | 0.000 | 0.000 |
| 34 | GSK3B | 0.342 | 0.008 | 0.485 |
| 35 | HIF1A | 0.222 | 0.001 | 0.442 |
| 36 | MAPK14 | 0.325 | 0.010 | 0.479 |
| 37 | MAOA | 0.393 | 0.036 | 0.507 |
| 38 | ABCC1 | 0.137 | 0.001 | 0.413 |
| 39 | SELP | 0.000 | 0.000 | 0.000 |
| 40 | PPARA | 0.179 | 0.001 | 0.383 |
| **No.** | **Protein Name** ^a^ | **Degree centrality (*C_d_*)** | **Betweenness centrality (*C_b_*)** | **Closeness centrality (*C_c_*)** |
| 41 | PPARD | 0.205 | 0.001 | 0.437 |
| 42 | PPARG | 0.239 | 0.004 | 0.448 |
| 43 | SELE | 0.068 | 0.000 | 0.377 |
| 44 | TYR | 0.308 | 0.012 | 0.466 |
| 45 | LCK | 0.085 | 0.000 | 0.337 |
| 46 | VDR | 0.060 | 0.000 | 0.329 |
| 47 | XDH | 0.248 | 0.000 | 0.390 |
| 48 | HPGD | 0.060 | 0.000 | 0.320 |
| 49 | HSD17B7 | 0.265 | 0.002 | 0.431 |
| 50 | ALDH2 | 0.256 | 0.000 | 0.392 |
| 51 | ALDH1A1 | 0.248 | 0.000 | 0.390 |
| 52 | AHR | 0.000 | 0.000 | 0.000 |
| 53 | ATXN2 | 0.000 | 0.000 | 0.000 |
| 54 | ABCG2 | 0.051 | 0.000 | 0.345 |
| 55 | MAPK10 | 0.385 | 0.014 | 0.503 |
| 56 | CALM1 | 0.248 | 0.004 | 0.451 |
| 57 | ABCC2 | 0.051 | 0.000 | 0.345 |
| 58 | CA1 | 0.026 | 0.000 | 0.250 |
| 59 | CA12 | 0.026 | 0.000 | 0.250 |
| 60 | CA9 | 0.026 | 0.000 | 0.250 |
| 61 | CBR1 | 0.265 | 0.000 | 0.394 |
| 62 | CBR3 | 0.265 | 0.000 | 0.394 |
| 63 | CCNB2 | 0.094 | 0.000 | 0.337 |
| 64 | CYP1A2 | 0.265 | 0.000 | 0.394 |
| 65 | CYP1B1 | 0.274 | 0.010 | 0.454 |
| 66 | CYP2A6 | 0.265 | 0.000 | 0.394 |
| 67 | CYP2B6 | 0.265 | 0.000 | 0.394 |
| 68 | CYP2C19 | 0.308 | 0.008 | 0.466 |
| 69 | CYP2C9 | 0.308 | 0.008 | 0.466 |
| 70 | HSD17B10 | 0.248 | 0.000 | 0.390 |
| 71 | HSD17B1 | 0.256 | 0.002 | 0.428 |
| 72 | HSD17B2 | 0.265 | 0.002 | 0.431 |
| 73 | GLO1 | 0.000 | 0.000 | 0.000 |
| 74 | GNAS | 0.436 | 0.069 | 0.538 |
| 75 | KMT2A | 0.060 | 0.000 | 0.320 |
| 76 | SELL | 0.000 | 0.000 | 0.000 |
| 77 | KDM4E | 0.000 | 0.000 | 0.000 |
| 78 | MAPT | 0.000 | 0.000 | 0.000 |
| 79 | MAPK1 | 0.453 | 0.028 | 0.534 |
| 80 | NFE2L2 | 0.000 | 0.000 | 0.000 |
| 81 | ABCB1 | 0.137 | 0.008 | 0.418 |
| 82 | RXRA | 0.316 | 0.022 | 0.485 |
| 83 | PIM1 | 0.128 | 0.000 | 0.411 |
| **No.** | **Protein Name** ^a^ | **Degree centrality (*C_d_*)** | **Betweenness centrality (*C_b_*)** | **Closeness centrality (*C_c_*)** |
| 84 | NEU2 | 0.000 | 0.000 | 0.000 |
| 85 | NR5A1 | 0.000 | 0.000 | 0.000 |
| 86 | SMN1 | 0.000 | 0.000 | 0.000 |
| 87 | UGT1A1 | 0.265 | 0.000 | 0.394 |
| 88 | UGT1A10 | 0.265 | 0.000 | 0.394 |
| 89 | UGT1A8 | 0.265 | 0.000 | 0.394 |
| 90 | UGT1A4 | 0.265 | 0.000 | 0.394 |
| 91 | UGT2B15 | 0.265 | 0.000 | 0.394 |
| 92 | TNKS | 0.000 | 0.000 | 0.000 |
| 93 | PIK3R1 | 0.376 | 0.010 | 0.499 |
| 94 | NOX4 | 0.000 | 0.000 | 0.000 |
| 95 | MAPK3 | 0.453 | 0.028 | 0.534 |
| 96 | TNKS2 | 0.000 | 0.000 | 0.000 |
| 97 | KDM4A | 0.000 | 0.000 | 0.000 |
| 98 | KDM4C | 0.000 | 0.000 | 0.000 |
| 99 | ALPI | 0.248 | 0.000 | 0.390 |
| 100 | SENP1 | 0.000 | 0.000 | 0.000 |
| 101 | VCP | 0.000 | 0.000 | 0.000 |
| 102 | CSNK1E | 0.128 | 0.000 | 0.356 |
| 103 | PTPN1 | 0.103 | 0.000 | 0.340 |
| 104 | GSK3A | 0.068 | 0.000 | 0.392 |
| 105 | JAK2 | 0.222 | 0.002 | 0.442 |
| 106 | PDPK1 | 0.291 | 0.003 | 0.401 |
| 107 | HSP90AA1 | 0.265 | 0.002 | 0.457 |
| 108 | HDAC1 | 0.248 | 0.005 | 0.451 |
| 109 | HDAC3 | 0.145 | 0.000 | 0.418 |
| 110 | PIN1 | 0.000 | 0.000 | 0.000 |
| 111 | STK11 | 0.171 | 0.000 | 0.371 |
| 112 | PARP1 | 0.000 | 0.000 | 0.000 |
| 113 | PRKCD | 0.162 | 0.000 | 0.379 |
| 114 | EGFR | 0.333 | 0.007 | 0.482 |
| 115 | PRKCA | 0.436 | 0.036 | 0.538 |
| 116 | P4HB | 0.000 | 0.000 | 0.000 |
| 117 | SI | 0.248 | 0.000 | 0.390 |
| 118 | MGAM | 0.239 | 0.000 | 0.388 |

a, Names of the target proteins are uniformed by Uniprot.
